# Supplementary material for: Optimizing Trial Designs for Targeted Therapies
Source: PLoS One. 2016 Sep 29;11(9):e0163726. doi: 10.1371/journal.pone.0163726 (PMC5042421; doi:10.1371/journal.pone.0163726)
Supplement: S1 File — (PDF) [file pone.0163726.s001.pdf]

Supplementary Material for Optimizing Trial Designs for Targeted Therapies

Thomas Ondra, Sebastian Jobjörnsson, Robert A. Beckman,
Carl-Fredrik Burman, Franz König, Nigel Stallard, and Martin Posch

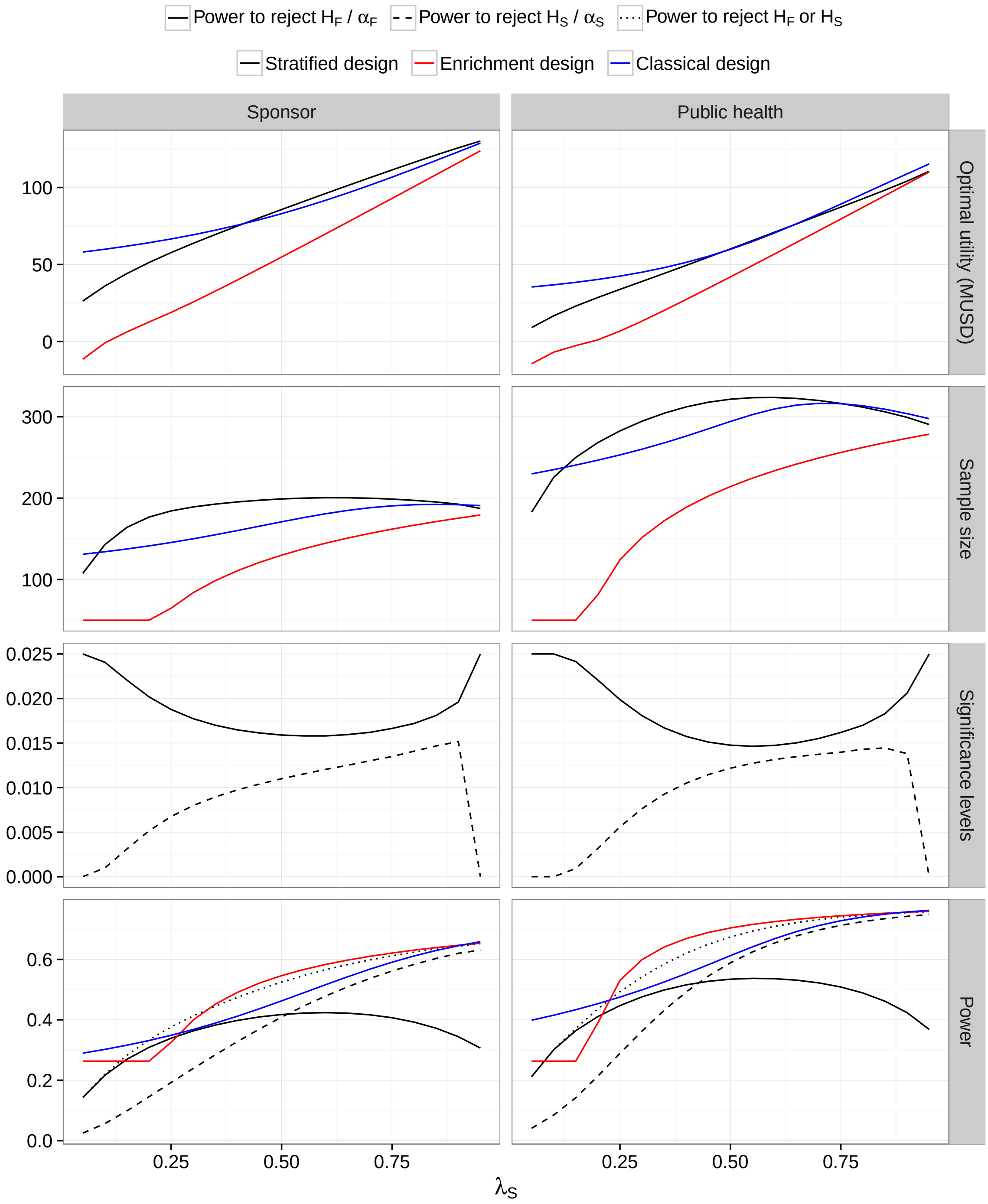

Fig A: Weak biomarker prior and a small market with biomarker costs (Case 3). Optimized expected utilities and sample sizes for the enrichment, classical and stratified design as function of the prevalence for $\lambda_S \in [0.05, 0.95]$. For the stratified design, optimized levels α_S and α_F for the multiple testing procedure are given. The last row shows the overall probability (averaged over the prior) that a significant treatment effect in H_S or H_F can be shown (and, for the stratified design, that the thresholds τ_S and $\tau_{S'}$ are crossed).

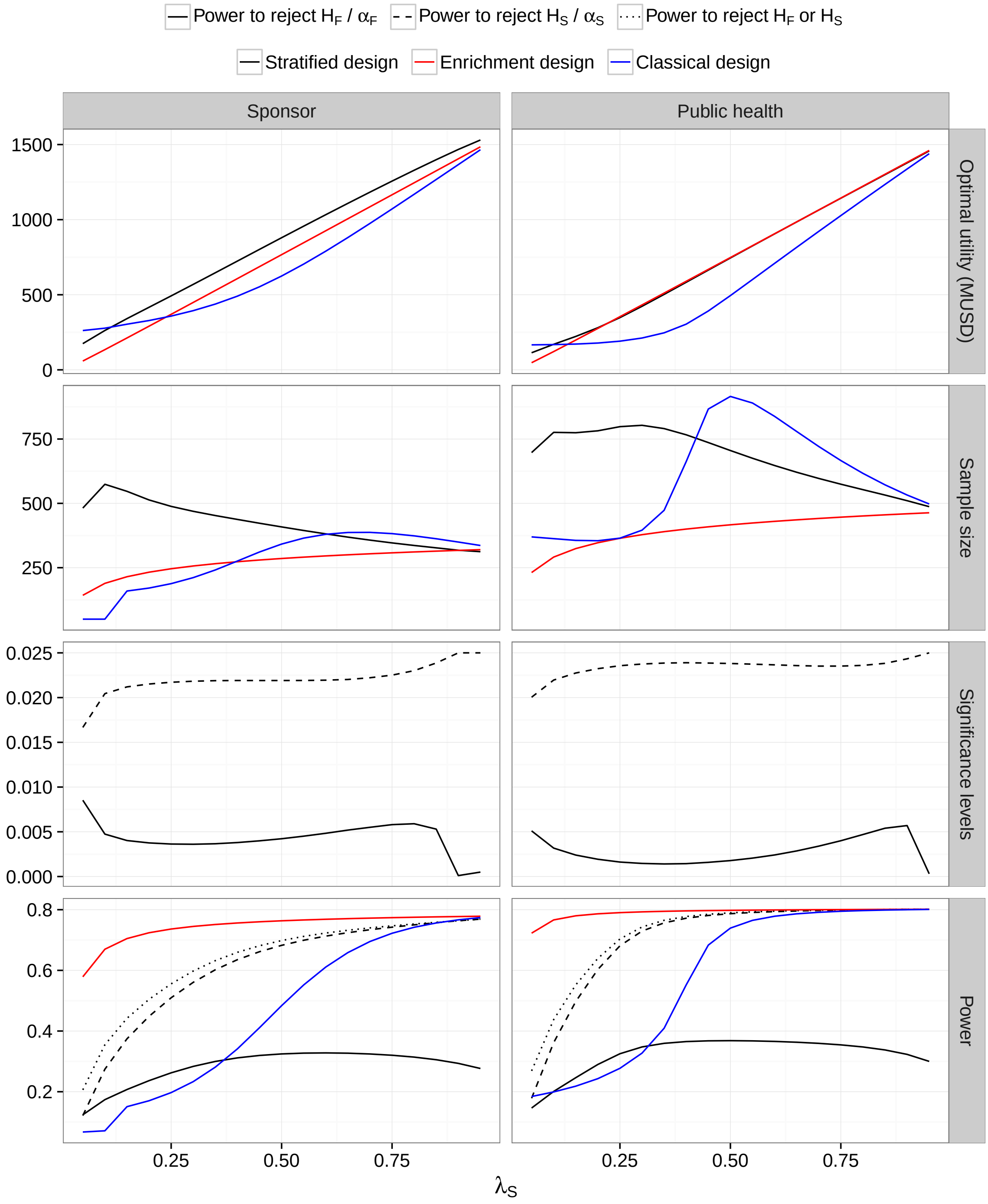

Fig B: Strong biomarker prior, large market and no biomarker costs (Case 1). See the legend of Fig A.

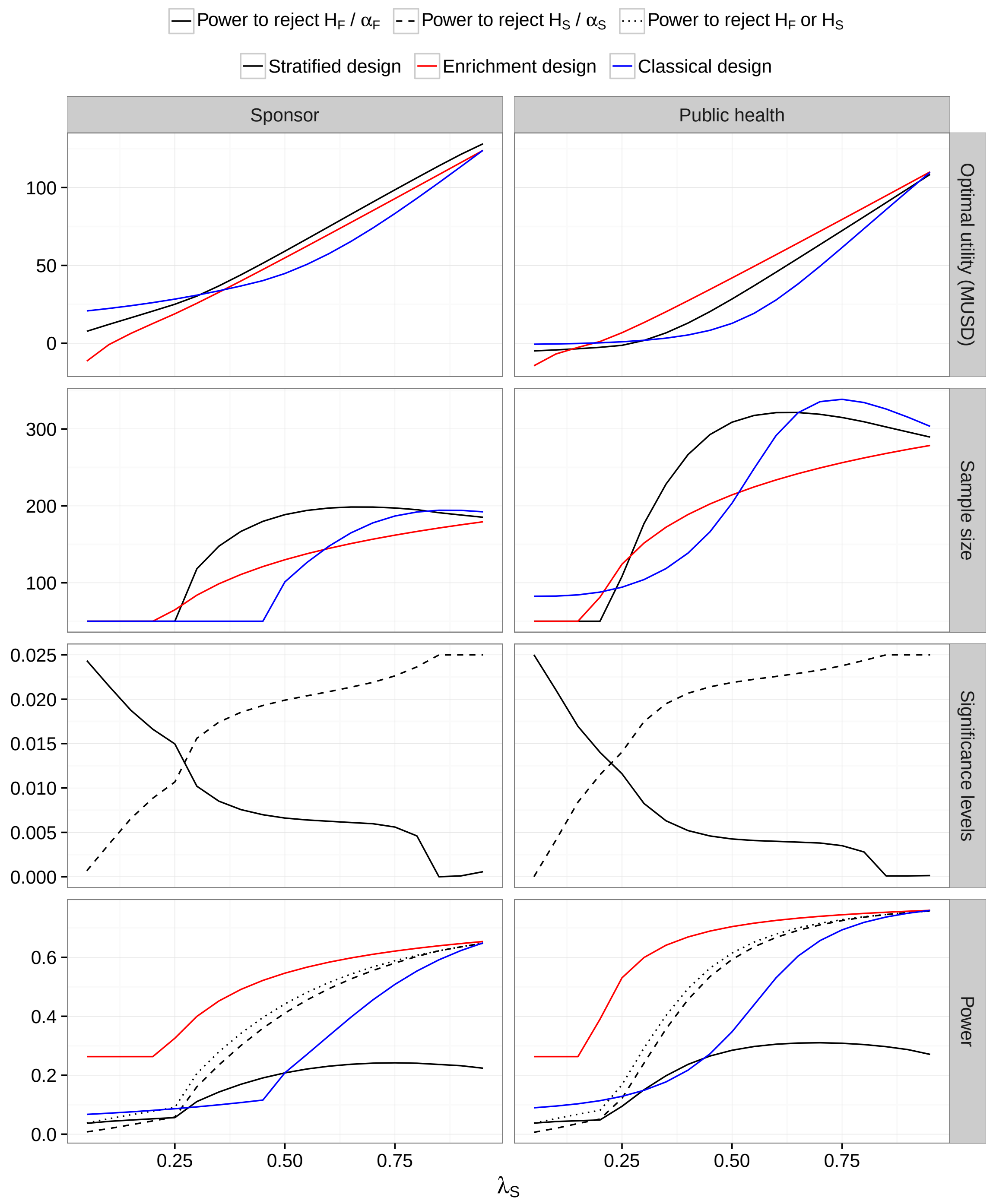

Fig C: Strong biomarker prior, small market with biomarker costs (Case 3). See the legend of Fig A.
